# Integration of primary care and palliative care services to improve equality and equity at the end-of-life: Findings from realist stakeholder workshops

**DOI:** 10.1177/02692163241248962

**Published:** 2024-05-11

**Authors:** Sarah Mitchell, Nicola Turner, Kate Fryer, Justin Aunger, Jude Beng, Emilie Couchman, Isabel Leach, Joanne Bayly, Clare Gardiner, Katherine E Sleeman, Catherine J Evans

**Affiliations:** 1 Division of Primary Care, Palliative Care and Public Health, Leeds Institute of Health Sciences, University of Leeds, Leeds, UK; 2 University of Nottingham School of Health Sciences, University of Nottingham, Queen’s Medical Centre, Nottingham, UK; 3 Academic Unit of Primary Medical Care, University of Sheffield, Sheffield, UK; 4 NIHR Midlands Patient Safety Research Collaboration, Murray Learning Centre, University of Birmingham, Birmingham, UK; 5 Health Sciences School, University of Sheffield, Sheffield, UK; 6 St Barnabas Hospices, Worthing, UK; 7 Cicely Saunders Institute of Palliative Care, Policy & Rehabilitation, Florence Nightingale Faculty of Nursing, Midwifery and Palliative Care, Kings College London, London, UK; 8 Sussex Community NHS Foundation Trust, Crawley, UK

**Keywords:** Palliative care, terminal care, health care facilities, manpower, services, general practice, primary care, realist research

## Abstract

**Background:**

Inequalities in access to palliative and end of life care are longstanding. Integration of primary and palliative care has the potential to improve equity in the community. Evidence to inform integration is scarce as research that considers integration of primary care and palliative care services is rare.

**Aim:**

To address the questions: ‘how can inequalities in access to community palliative and end of life care be improved through the integration of primary and palliative care, and what are the benefits?’

**Design:**

A theory-driven realist inquiry with two stakeholder workshops to explore how, when and why inequalities can be improved through integration. Realist analysis leading to explanatory context(c)-mechanism(m)-outcome(o) configurations(c) (CMOCs).

**Findings:**

A total of 27 participants attended online workshops (July and September 2022): patient and public members (*n* = 6), commissioners (*n* = 2), primary care (*n* = 5) and specialist palliative care professionals (*n* = 14). Most were White British (*n* = 22), other ethnicities were Asian (*n* = 3), Black African (*n* = 1) and British mixed race (*n* = 1). Power imbalances and racism hinder people from ethnic minority backgrounds accessing current services. Shared commitment to addressing these across palliative care and primary care is required in integrated partnerships. Partnership functioning depends on trusted relationships and effective communication, enabled by co-location and record sharing. Positive patient experiences provide affirmation for the multi-disciplinary team, grow confidence and drive improvements.

**Conclusions:**

Integration to address inequalities needs recognition of current barriers. Integration grounded in trust, faith and confidence can lead to a cycle of positive patient, carer and professional experience. Prioritising inequalities as whole system concern is required for future service delivery and research.

**What is already known about the topic?**

- Almost one-quarter of people who could benefit from palliative and end of life care do not receive it, and this is worse for people who live in the most socioeconomically deprived and ethnically diverse areas.
- Integration of primary care and specialist palliative care services has the potential to address current inequality in access to community palliative care and high quality, personalised care at the end of life.
- Integrated approaches to palliative and end of life care are under-researched but the evidence suggests that care is poorly coordinated, especially for people near the end of life and that identification of patients with cancer who could benefit from palliative care is better than for those with non-cancer conditions.

**What this paper adds?**

- Patients and carers from minoritised communities experience cultural norms, power imbalances and racism that preclude them from accessing current primary care and palliative care services.
- Key factors in the delivery of co-ordinated care and continuity are trusted interpersonal relationships, faith in the approach and confidence in the system.
- Shared vision and professional commitment to addressing inequalities in palliative and end of life care is an important contextfor change, triggering mechanisms that lead to beneficial outcomes at every system level, from interpersonal and team relationships, to organisations.

**Implications for practice, theory or policy**

- Future integration of primary care and specialist palliative care should be tailored to different contexts and communities to avoid ‘one size fits all’ services which are inaccessible to people from minoritised communities.
- Positive patient experiences grow the confidence of the multi-disciplinary team and drive commitment to efforts to deliver more equitable palliative care, producing a ripple effect that can affirm the efforts of stakeholders at each level of the health and care system.
- Improving equitable care requires recognition of the barriers that currently exist and the adoption of allyship as a continual learning process at all health and care system levels, from interpersonal relationships to system leadership.

## Background

Population need for community palliative and end of life care is rising, with aging, frailty, rising health-related suffering and increasing numbers of people dying at home.^1,2^ Access to good community palliative and end of life care is inconsistent and there are longstanding inequalities (unfair and avoidable differences) in access to specialist palliative care services for people with non-cancer disease, from different ethnic groups and areas of low socioeconomic status.^3–5^ Patients from areas of high socioeconomic deprivation, and those from Black and minority ethnic backgrounds are less likely to receive such care.^4–8^ Specialist palliative care services, including hospices, are not consistently available and do not have the resource or capacity to provide care for all dying people. Palliative care is an essential and critical function of primary care internationally but approaches to generalist palliative care in primary care are highly variable.^9,10^ Table 1 provides a description of primary care, generalist and specialist palliative care, and how these interact.

**Table 1.** Understanding aspects of primary palliative care.

Speciality	Description
Primary care	The first point of contact for healthcare in the community for people seeking health advice and treatment. Primary care is person-centred rather than disease-centred and focusses on people’s needs. Primary care provides care ranging from disease prevention and treatment, through to rehabilitation and palliative care. This is delivered by a wide range of professionals including community nursing services, opticians, pharmacists and dentists. General practice is the medical speciality aligned to primary care.
Generalist palliative care	Holistic, person-centred care, focussed on quality of life for people with advanced disease and their carers. This is provided by their usual care team. In the community, this may include general practitioners, district and community nurses, pharmacists, physiotherapists, occupational therapists and social carers.
Specialist palliative care	Specialist palliative care is delivered by a multidisciplinary team (MDT) of staff with the qualifications, expertise and experience in palliative care. Specialist palliative care is required by people with progressive life-limiting illness, with complex needs that cannot be addressed through the care and capability of their usual care team.

Identification of palliative care needs, described as a ‘golden ticket’ to enhanced care in the community, is lacking, particularly for people with non-malignant disease.^3^ Emergency hospital admissions rise towards the end of a person’s life, and can be burdensome and unwanted. Patients from areas of socioeconomic deprivation are more likely to be admitted to hospital towards the end of life because they are more unwell and may be less well placed to cope with the end of life at home.^6^ A key characteristic of good community palliative and end of life care is continuity of care with primary care teams, which is associated with less frequent emergency healthcare use towards the end of life for patients of all ages.^7–10^. However, overstretched primary care services are struggling to prioritise palliative and end of life care due to time pressures, compromised continuity (including out-of-hours), inconsistent training, skills and confidence, and variable access to specialist palliative care services.^11–13^

Integration between primary care and specialist palliative care is a potential way to improve quality and address inequalities in community palliative and end of life care.^14,15^ Integration describes health and care services working together so that the care received by an individual is co-ordinated, personalised and seamless. Efforts to integrate effectively are required in interpersonal relationships, team interactions and organisation and health and care system levels, as outlined in the levels of integration in Table 2.^16^

**Table 2.** Conceptualisation and levels of integration.

Type of integration	Levels of the healthcare system	Definition
Integrated care	Interpersonal	Care that meets the personal needs of an individual in an efficient way.
Clinical integration	Organisation/team	The coordination of care into a single and coherent process, either within or across professions for example, through shared guidelines or protocols across organisational boundaries.
Service integration	Organisation/team	Co-ordinated working across different services for example, through a cross-organisational, multi-disciplinary team or a single referral processes.
Organisational integration	Organisation/health and care system	The bringing together of coordinating structures and governance across organisations for example, in organisational mergers or through contractual or cooperative arrangements.
Administrative or functional integration	Organisation/health and care system	Where non-clinical support and back-office functions are joined up, or data is shared, across organisations.

### Study rationale and subject of inquiry

The delivery of palliative and end of life care through integration of primary care and specialist palliative care is under-researched and urgently needed to inform new integrated models of community palliative care, to meet rising need and address inequality.

### Research questions

This realist study addresses the questions: ‘how can inequalities in access to community palliative and end of life care be improved through the integration of primary and palliative care, what are the benefits, and for whom?’

### Initial programme theory

Programme theory of effective integration in healthcare was identified in the work of Aunger et al.^17^ This theory of collaboration in healthcare describes (i) entry into partnership, (ii) partnership functioning and (iii) partnership effectiveness leading to partnership synergy (or not).^18^

## Methods

### Rationale for a realist study

The effective delivery of palliative and end of life care is complex because it depends on the active input of individuals, including patients, specialists and non-specialists, embedded in social infrastructures and influenced by wider, organisational and cultural factors.^19,20^ When multiple organisations enter a process of collaboration to deliver integrated care, the complexity increases.^21^ Realist research is theory-driven, explanatory and suitable for the study of complex healthcare interventions and systems (‘programmes’). Realist methodology assumes that the same intervention will not work in the same way for everyone, everywhere, because non-observable influences (such as culture and politics) have an effect. This methodology is valuable because it considers the relevant contextual factors (‘when?’), underpinning hidden mechanisms (‘how?’ and ‘why?’) and ‘for whom?’ the process of integration produces beneficial outcomes (‘works’).

The aim of realist research is to generate transferable theory about how a particular intervention may, or may not, work in particular circumstances, for certain people. The research questions were therefore addressed by gathering insights and conducting analysis to propose contexts (C) in which the integration of primary care and specialist palliative care services is most effective, the outcomes (O) achieved and the underlying, hidden mechanisms (M) (changes in reasoning, emotional responses and behaviour) that are triggered in certain contexts to lead to these outcomes. New theory is generated through a series of context-mechanism-outcome configurations (CMOCs).^20^

### Study design

Two realist workshops were held with key stakeholders. Workshop 1 focussed on factors that affect integration between primary care and specialist palliative care. Workshop 2 considered integration to address inequalities. This study was part of a wider National Institute for Health Research Palliative Care Partnership project: REducing inEQUalities through integration of Primary and Palliative Care (RE-EQUIPP). The partnership aimed to deliver virtual research and practice workshops with multi-disciplinary collaborators across priority areas of (1) integration and (2) inequalities, to generate new insights, understanding and build theory to inform future research. The partnership plan is published online.^22^

### Recruitment

Recruitment occurred via a snowballing approach through professional networks. The aim was to recruit participants with relevant experience from primary care, specialist palliative care, patient and public involvement members, commissioners and others from social care, the voluntary sector, healthcare and academia. Workshop participants from Black and minority ethnic backgrounds were proactively recruited, particularly patient and public involvement members.

### Data collection

The workshops were conducted remotely via an online platform (Zoom). A realist topic guide, informed by recent research, provided structure (Appendix 1).^23^ Workshops were audio-recorded, and field notes kept. Recordings were transcribed verbatim and anonymised by a university-approved transcription provider.

### Data analysis

Data analysis was informed by the initial programme theory^17^ and conceptualisation of integration.^16^ Reflection and note-taking began alongside data collection. Workshop transcripts and field notes were read and re-read. Every section of text was colour-coded according to whether it alluded to a context (C), mechanism (M) or outcome (O) by two researchers (SM and NT). All sections of text that pertained to a particular issue related to the delivery of integrated palliative care across each level of integration were extracted into a bespoke data extraction table, manually coded and organised into overarching themes (examples are provided in Supplemental File 1). Explanatory CMOCs were proposed, then refined and refuted through discussion with the research team (SM, CJE, KES, NT, IL, KF, EC, JA) which included identifying patterns in the data, retroduction (inductive reasoning to derive new theory from multiple observations) and deductive logic (testing ideas against existing theory).^24^

## Findings

### Details of participants

A total of 27 participants attended two online workshops in July and September 2022: patient and public members (*n* = 6), commissioners (*n* = 2), clinicians and researchers from primary care (*n* = 5) and specialist palliative care (*n* = 14). Most were White British (*n* = 22), other ethnicities were Asian (*n* = 3), Black African (*n* = 1) and British mixed race (*n* = 1).

### Realist findings

Contexts are labelled ‘(C)’, mechanisms ‘(M)’ and outcomes ‘(O)’. For clarity, numbers have been applied to link each CMOC. Illustrative quotes from the workshops are included. A considerable amount of the data from the workshops related to current contexts. CMOCs were formulated by prioritising the description of beneficial outcomes for patients and carers, achieved in certain contexts (or not) through specific mechanisms. The following CMOCs are aligned to the stages of partnership described in the initial programme theory, (i) entry into partnership, (ii) partnership functioning and (iii) partnership effectiveness.^18^

#### Patient and carer experience of the current context

Patient and carer participants described awareness of the incurable nature of their conditions and a sense that their palliative care needs were not met through current services, because, due to their ethnic or cultural background they are ‘different’ and ‘do not fit the mainstream’. They described a perception that they needed ‘specific language’ or a certain way of communicating to express such needs and effectively navigate the norms of the current healthcare system (C1). Without this they could not access the services that they needed, resulting in a lack of confidence that their needs could or would be met (M1). Participants described the need to make a choice to ‘look after themselves’ instead (O1):

*‘As a Muslim, in the present climate, you’re very reluctant to ask for things, or to be seen as causing trouble, so it’s quite difficult to go into a health situation and say actually, this doesn’t meet my needs, I would need you to do it like this, that or the other’* [Researcher, Workshop 2].

Systemic power imbalances and racism had been experienced by participants and were a pervasive barrier to accessing care (C2):

*‘There is a massive issue with racism . . . if we don’t actually get a grip on this, we’re also going to have a lot more problems in regards to actually being able to offer any form of cultural competency in care’* [PPI member, Workshop 2]

A need to comply with certain cultural norms and fit into a ‘one size fits all’ approach to palliative care hindered people from different cultures accessing services. Participants described the need to be a ‘good patient’, who did not speak up (M2), but this caused mistrust in services and the system (O2):

*‘Because within different cultures, there are different ways of doing things, aren’t there. But in medicine, and healthcare, we love to make sweeping generalisations, and do a one size fits all model’* [Primary Care Professional, Workshop 2]

The lack of diversity in the workforce, particularly in specialist palliative care, was recognised as a factor that contributed to the current context.

*‘a very white, very middle-class profession and there’s an obvious knock-on from that’* [Palliative care professional, Workshop 2]

**Figure 1.**
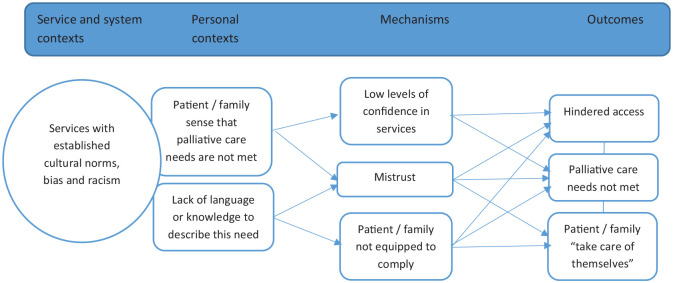
CMOCs to explain the current context with barriers and enablers to culturally competent palliative care (CMOCs 1–2).

The CMOCs reflecting experiences of patients and carers in the current system are summarised in Figure 1:

#### Entry into integrated palliative care partnerships

Inequalities in palliative care were acknowledged as a whole health and social care system challenge, with the potential to galvanise professionals, teams and services to work differently. A recent example of responsive change to increased need occurred during the COVID-19 pandemic, when rapid changes in service delivery were imperative as the need for community end of life care rose suddenly (C3). Shared concerns amongst frontline professionals and leaders of services and systems led to clarity and vision, which stimulated a shared commitment (M3) to work in new ways to deliver care (O3). Decreased bureaucracy, with health services in ‘crisis response’ (C4), provided a sense that longstanding barriers to integrated working were suspended and there was freedom to innovate (M4). Cross-boundary solutions to meet population need were developed quickly (O4).

As well as shared commitment and vision, and space to innovate, organisational and system leadership was described as an important facilitator or barrier to change. Leaders require personal commitment to addressing inequalities in palliative care (C5). System leaders are role-models. Improving diversity in leadership, and leaders adopting allyship as an anti-racist approach can influence others (M5) and could lead to the development of more culturally competent palliative care in the future, especially if there is leadership of activities such as co-design (O5), where the views of patients and carers are heard: .

*‘And also making sure that in any discussions at the top about strategy around palliative care, it’s making sure that we have commissioners and carers, both those in a professional sense but also those in a family and friend sense, at the table as well as voices of those that we don’t always hear’* [PPI member, Workshop 2]

**Figure 2.**
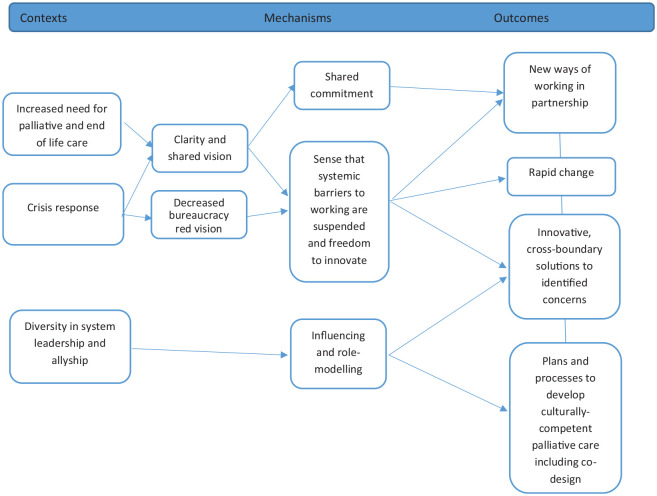
Entry into integrated primary and palliative care partnerships (CMOCs 3–5).

The CMOCs to describe entry into partnership through response to a whole health and care system challenge, and leadership, are outlined in Figure 2:

#### Effective partnership functioning to deliver integrated palliative care and address inequalities

Participants acknowledged the multi-disciplinary team required for palliative care, who, from a patient perspective, must work together to deliver integrated care as described below:

*‘patients wouldn’t actually notice the integration, that’s the whole point of it should be that to the patient it just. . . patients, they don’t tend to distinguish between services anyway, but I think that they would not distinguish between services, and they would be satisfied would probably be the outcome of really good integration’* [PPI member, Workshop 2].

An identified individual from the team (C6) who co-ordinates, proactively advocates and takes responsibility for care delivery (M6), is valued by patients and families (O6). This individual could be a general practitioner, community specialist palliative care nurse, district nurse, an allied healthcare professional or a social prescriber, but the nature of the relationship, with accessibility and trust, is key:

*‘A professional who can coordinate and communicate and collaborate well and do things, it will at least solve one of their so many problems they are going through’* [PPI member, Workshop 1]

To deliver culturally competent care, the co-ordinating professional requires commitment to understanding culture and religion beyond their own (C7), and an ability to reconcile limitations in their understanding and knowledge (M7). Sharing this openly and honestly with patients and carers is appreciated and helps to build understanding and trust (O7). For example, in the quote below, the participant explained the need for washing rituals and dietary requirements related to medication. If professionals had recognised and appreciated these, it would have enhanced compliance:

*‘None of that engagement took place [about individual holistic needs] . . . If they had actually understood the overall needs, the dietary needs of the patient, the behavioural needs of the patient, perhaps the medication would have been [effective]’* [PPI member, workshop 2]

Models of palliative care that include the provision of care by people from within communities were described. Mutual previous experiences (C8) can provide an unspoken understanding of a situation, and increase confidence amongst patients and carers (M8), enabling sensitive discussions about palliative care, death and dying (O8):

*‘It really, really helped that she [healthcare professional] just walked into our house and immediately understood that we were probably doing things slightly differently [because of our cultural background]’* [Researcher, Workshop 2]

**Figure 3.**
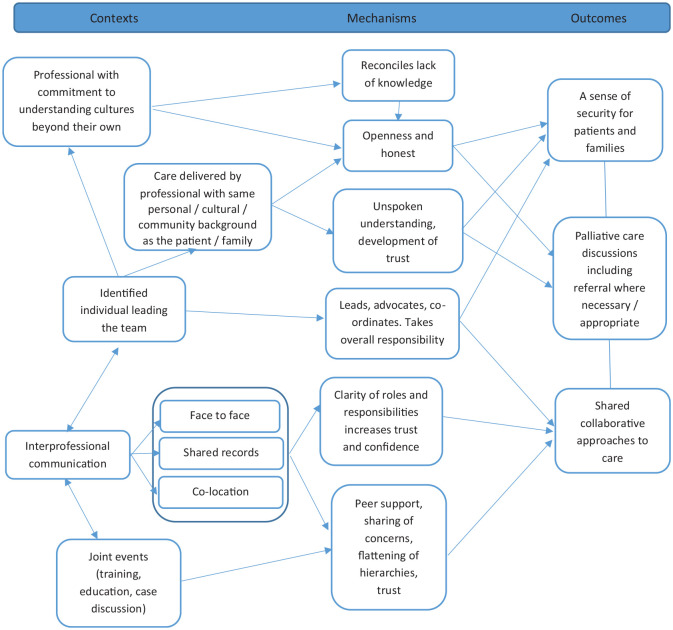
Effective professional and service partnership functioning to deliver integrated palliative care (CMOCs 5–10).

A lack of clarity around roles and responsibilities of multi-disciplinary team members (C9) can lead to incorrect assumptions (M9), and tense or fragile relationships (O9). Communication is a key factor driving partnership effectiveness. Opportunities for team members from different organisations to work together in the same geographical location was suggested as context (C10) to enhance communication and team dynamics (O10), allowing trust and confidence to build (M10) through informal discussions, sharing clinical concerns and learning and peer support. Joint case discussions, training and education are also valued (C11). These events bring teams together in a way that can flatten professional hierarchies, improve understanding of perspectives (M11) and lead to the development of collaborative approaches to a shared issue or concern (O11). Digital enablers were discussed, including online team meetings. Inconsistent electronic medical record sharing for palliative care was identified as an important limiting factor in terms of clinical, service and organisational integration. Figure 3 summarises these CMOCs to outline professional and service partnership functioning to deliver integrated palliative care.

#### Achieving integrated palliative care through partnership synergy

CMOCs 1–10 provide a set of theoretical explanations about how entry into partnership, and effective partnership functioning, can produce beneficial outcomes. Trust is a prominent feature in each CMOC, building confidence and faith in the partnership collaboration and integrated approach. This is important for all stakeholders, including patients and carers, who describe benefits in nuanced palliative care consultations where uncertainties are shared (M11). This is enabled by time with professionals and continuity (C11), leading to a sense of security, faith and confidence (O11), as described in the quote below:

*‘as a patient you should almost not notice it’s going on and that would signify that it’s a successful collaboration . . . the other benefits are kind of further down the line and for the professionals involved I think as well, which ultimately benefit the patients in the end as well*’ [PPI member, Workshop 2]

**Figure 4.**
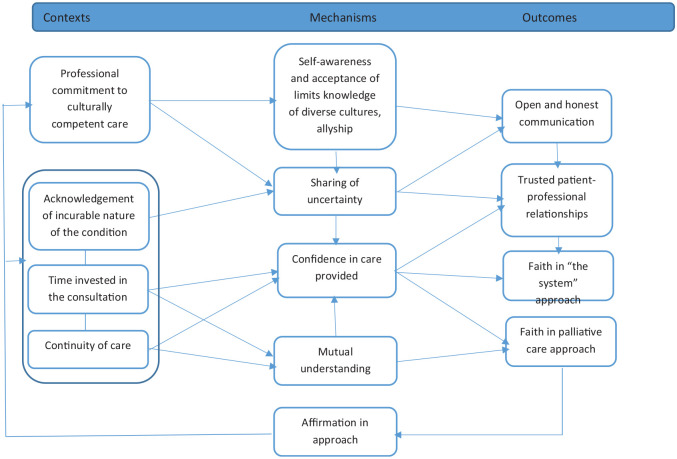
Partnership functioning and synergy (CMOCs 11).

Positive experiences of patient care affirm and build confidence in team relationships and supporting systems, leading to partnership synergy. Key contexts for this include professional commitment at interpersonal, organisation and systems levels (C12) with self-awareness and allyship (M12) leading to trusted relationships with patients (O12). Provision of care in this integrated way drives commitment to similar work in the future (O12), as outlined in Figure 4:

*‘[this way of working] builds our trust and enables us to do it again as a team the next time’* [Palliative Care Professional, Workshop 2]

## Discussion

### Main findings

This realist study provides new insights from key stakeholders, including patient and public involvement members, with the potential to improve inequalities in access to community palliative and end of life care through the integration of primary and palliative care. Current inequalities in palliative and end of life care are a whole health and care system concern. Increased recognition of these inequalities could and should mobilise efforts to develop innovative, cross-boundary integrated approaches to palliative care. As new integrated models of care are developed, the power imbalances, cultural norms, racism and limitations of a ‘one size fits all’ service should be challenged so that people from minoritised backgrounds benefit.

In multi-organisation, multi-professional teams, clarity of roles and effective communication enable trust and confidence. Co-location of professional teams, reliable record sharing and working towards a shared goal are enablers. Effective integrated palliative and end of life care is well co-ordinated, with professionals willing to take responsibility and commit to understanding cultures beyond their own.

#### Strengths and limitations

The strength of the realist approach is its explanatory nature. This study provides early insights into effective integration across primary and palliative care services. The theoretical framework and programme theory informing the analysis adds to the study’s strengths and potential applicability of the findings in future research and service design. The study is small however, with only 27 participants in two workshops. Efforts were made to recruit people from diverse ethnic, cultural and socioeconomic backgrounds, but there were notable gaps in representation of people from other marginalised societal groups. Much more needs to be done in future studies to ensure diversity both within the research team and amongst participants. Organisational and system factors that lead to effective integration are important areas for future research.

Although neither a realist review nor evaluation, this paper has adhered to the RAMESES publication standards where possible.^25^

#### What this study adds?

Although small, this study contributes to the evidence to support the development of integrated palliative and end of life care, which is currently under-researched. To date, Very few published studies investigate integrated approaches to care that involve primary care and palliative care services, and consider inequalities.^14,26–28^ The findings of this study are in keeping with the findings of this limited previous research which suggest poor identification of patients with palliative care needs and poorly co-ordinated care but provide little insight into the reasons for inequalities of access to palliative care for people from different societal groups and communities. This study provides powerful new insights into patient and carer experiences of racism and cultural norms that preclude them accessing services. Shared vision and commitment to addressing inequalities in palliative and end of life care is required at every level of health and care systems, from interpersonal and team relationships to organisation and system leadership. Identifying that racism exists in palliative care, and adopting allyship as a continual learning process, are important solutions and areas for future research.^29^

Integration between palliative care specialists, primary care and community nursing teams provides valuable continuity and a means of managing or escalating difficult symptoms.^14^ In a previous study (led by a member of this team), a short-term integrated model of palliative care for older adults with noncancer conditions was effective and cost effective.^14^ This study suggests that key factors in the delivery of co-ordinated care and continuity are trust and confidence in patient-professional relationships. This is in keeping with the previous research, where enablers included (1) established relationships between the primary care and specialist palliative care team members, (2) community nurses and general practitioners being recognised as the main generalist healthcare providers of palliative and end of life care and (3) their involvement in the development of the model of care. Pervasive challenges include inconsistent health record sharing, including handover information from out-of-hours contacts.^26,28,30^ Infrastructure for shared records, as well as shared work environments, teaching and peer support, were all identified as important in this study. The findings of this study are also in keeping with previous realist research suggesting that functioning partnerships, where benefits are perceived by stakeholders, can affirm efforts, producing a ripple effect that enables further performance benefits.^31^ Positive patient experiences provide affirmation for the multi-disciplinary team, growing confidence and driving commitment to efforts to deliver more equitable palliative care in partnership.^17^

Compromised trust, faith and confidence, for example when an investment in resources does not result in the desired effects, can result in partnership inertia and breakdown. The COVID-19 pandemic was a time during which innovative, integrated approaches were developed quickly, with a shared goal and decreased bureaucracy.^21^ Beyond the pandemic, learning should be maintained and considered carefully as policy advocates for more integrated models of primary care. More integrated primary care and palliative care research is necessary to accompany service developments, providing opportunity to ensure more palliative and end of life care for under-served communities.

## Conclusion

Effective integration of primary care and specialist palliative care to address inequalities depends on a recognition of the barriers that currently exist, including cultural norms and racism. New models of integrated care, grounded in trusted interpersonal relationships, faith in the approach and confidence in the system, could lead to better co-ordination and a cycle of positive patient, carer and professional experience, but research is required to understand how this works in different contexts, to avoid future ‘one size fits all’ services. Leadership and commitment to a shared vision that prioritises inequalities as whole system concern, will be required to drive innovative new service models and research.

## Supplemental Material

sj-docx-1-pmj-10.1177_02692163241248962 – Supplemental material for Integration of primary care and palliative care services to improve equality and equity at the end-of-life: Findings from realist stakeholder workshops Supplemental material, sj-docx-1-pmj-10.1177_02692163241248962 for Integration of primary care and palliative care services to improve equality and equity at the end-of-life: Findings from realist stakeholder workshops by Sarah Mitchell, Nicola Turner, Kate Fryer, Justin Aunger, Jude Beng, Emilie Couchman, Isabel Leach, Joanne Bayly, Clare Gardiner, Katherine E Sleeman and Catherine J Evans in Palliative Medicine

## Appendix

**Appendix 1.** Topic guides.

Workshop 1: integration.	
	Questions/prompts
Introductory questions to promote open discussion	What are your views on integration in palliative care *From the perspective of patients/primary care/specialist palliative care* *Is it possible?*
Partnership functioning Exploring contexts For example, established relationships, enabled by effective infrastructure, personal commitments, organisational commitment	When does ‘integration’ happen? *What circumstances are needed?* *What brings people/teams together?* *Are there factors that are essential? individual/organisational/policy factors?* *Can anyone describe examples or experiences of integration?* *How was the service developed? Why? Who was involved?* *Did it ‘work’? In what way did it work?* *What was it that made this work?*
Partnership behaviour Looking for mechanisms For example, trust, confidence, faith, shared vision, shared commitment, shared philosophy of care	*How* does integration happen? *How do we know when a service is ‘integrated’?* *What is it like to work in an integrated way?* *How does it feel for patients/clinicians?* *How is that different to other ways of working?* *What is needed at an organisational level?* *How could policy improve/support this?*
Partnership effectiveness Looking for known and perceived beneficial outcomes – and to explore how these may relate to outcomes outlined in policy as desirable – important outcomes for patients/family may differ to those outlined in policy Examining further possible outcomes – wider perceived benefits and/or unintended consequences	What do you think are the potential benefits for patients and families of integrated models *Does anyone else benefit from integration?* *For whom are there benefits?* *What are the positive outcomes?* *Are there any less positive outcomes?*
If time: thinking about future research.	How could we test or evaluate integration in palliative care?

**Table:** Workshop 2: inequalities.

	Questions/prompts
Addressing inequalities through identification of need Looking for mechanisms For example, trust, confidence, faith, shared vision, shared commitment, shared philosophy of care, appetite for innovation Exploring contexts For example, building on established relationships, enabled by effective infrastructure, organisational commitment, policy	How can identification of need be improved? How can integrated working improve this process? What information/support do patients need? Could innovation happen through integrated working? What kinds of innovation? What kind of policy could support this? What kind of research do we need? *Any examples or experiences of this?* *How was the service developed? Where? Why?* *Who was involved?* *Did it ‘work’? In what way did it work?* *What was it that made this work?*
Addressing inequalities in access to specialist services Exploring contexts For example, building on established relationships, enabled by effective infrastructure, organisational commitment, policy Looking for mechanisms For example, trust, confidence, faith, shared vision, shared commitment, shared philosophy of care, appetite for innovation	What are the causes for current inequalities in access to specialist services? How could these be overcome through integration? What would access to specialist services look like if there was true integration? How would that be experienced by patients and families?
Integrated services to meet need (partnership effectiveness and sustainability) Looking for known and perceived beneficial outcomes – and to explore mechanisms/important outcomes for patients/family	What would a truly integrated service look like from the perspective of patients and families? How would that be experienced? *What is needed at an organisational level?* *How could policy improve/support this?* *What research is needed?* *Does anyone else benefit from integration?* *For whom are there benefits?* *What are the positive outcomes?* *Are there any less positive outcomes?*
