# Supplementary material for: Integration of primary care and palliative care services to improve equality and equity at the end-of-life: Findings from realist stakeholder workshops
Source: Palliat Med. 2024 May 11;38(8):830–41. doi: 10.1177/02692163241248962 (PMC11448105; doi:10.1177/02692163241248962)
Supplement: sj-docx-1-pmj-10.1177_02692163241248962 – Supplemental material for Integration of primary care and palliative care services to improve equality and equity at the end-of-life: Findings from realist stakeholder workshops [file sj-docx-1-pmj-10.1177_02692163241248962.docx]

### Integration of primary care and palliative care services to improve cultural competency and equity at the end-of-life: CMOC development

1. *How* can the integration of primary and palliative care reduce inequalities in access to palliative and end-of-life care in the community?
2. What are the mechanisms through which integrated palliative care in the community is achieved?
3. What are the important contexts that determine whether or not these mechanisms lead to beneficial outcomes for patients and caregivers?
4. What are the implications for future research, policy and practice?

| **Data pertaining to context(s)** | **Data suggesting mechanisms** | **Data to describe outcomes** | **CMOC** |
| --- | --- | --- | --- |
| **INEQUALITIES** | | | |
| **RACISM**  If we don’t actually get a grip on this [racism] we're also going to have a lot more problems in regards to actually being able to offer any form of cultural competency in care.  there is a massive issue with racism,  Because within different cultures, there are different ways of doing things, aren't there.  But in medicine, and health care, we love to make sweeping generalisations, and do a one size fits all model  that you’re never going to understand everything and also people obviously vary as well within cultures. Not everybody has exactly the same beliefs or holds those cultural things as of equal importance | None of that engagement took place [about individual holistic needs], and it was only from the sort of understanding on how the health was not being delivered effectively. If they had actually understood the overall needs, the dietary needs of the patients, the behavioural needs of the patients, perhaps the medication would have been [effective]…  while we focus on medical and health needs, seeing the signs that that’s not working, that is really important.  Because we're not listening, and we don't want to listen.  And that may be, some of it is due to time, but a lot of it is due to, I don't want to know anything about this person, because this person is a number, and I need to move onto the next four or five people in this little bay, I need to deal with all of them  and people not wanting to understand.  It isn't about not understanding, it's about not wanting to understand. | I had a family member go over and say, she's Muslim, we need this, we need that, and the receptionist just looking and going, well get in line because everybody else has died as well.  when people feel they’ll treated as a person, as an individual, not a number, and that has come through quite a number of pieces of work that I’ve done that that theme about that individualisation and not just another person in the queue kind of thing. | Racism exists in the (at every level) NHS and in palliative care (c) and is a barrier to the provision of equitable, culturally competent care.  The delivery of culturally competent palliative care depends on professional commitment to understanding culture and religion beyond their own (m), and responding accordingly (m) in the delivery of care (o).  Understanding individual, holistic need includes all aspect of a person’s, not only medical and health needs (c). The effective delivery of interventions in palliative care, including medication, depends on an appreciation and understanding of how the person will (or will not) engage with the intervention (m) and the impact this has on their life (o). |
| **CULTURALLY COMPETENT CARE – SERVICE DELIVERY & AWARENESS/ACCEPTANCE OF SERVICES**  people of different cultures and backgrounds I've learned that what tends to happen is, people think, okay this service doesn't cater for our needs, so we need to look after this ourselves.  I think the automatic reaction will just be, well okay, we do that ourselves because these services aren't suitable for us.  the challenge with palliative care and end of life care is additional stigma, and perhaps, additional, then, cultural issues that may mean that people are less willing to talk about things  Not by giving the same information to all communities, it needs to be relevant to that particular community in terms of behaviour.  “The need for that highly personalised care”  My mother was a person, not just because of a generational issue, but because of the cultural issue, she will not take any form of medication, unless she has washed herself, she has made, prepared herself, and dressed herself, and felt that she is fully awake, and she is, you know, ready.  And then she will take the medication.  That’s just, you know, a cultural issue, maybe a generational issue, maybe a behavioural issue. | I think we have an idea of trying to be a good patient and to get the care that we need we have to be a good patient and we have to be quite well-behaved and stating preferences doesn’t fit into that, does it? Refusing something because it doesn’t fit with your cultural needs doesn’t really fit with the idea of being a good patient.  People are in the habit of not saying what they need  You need to probe into people's needs, you know, gently, but quite persistently.  As a Muslim, in the present climate. You're very reluctant to ask for things, or to be seen as causing trouble, So it's quite difficult to go into a health situation and say, actually, this doesn't meet my needs, I would need you to do it like this, that, or the other.  People would really need to be encouraged to describe what their needs are, not just given a piece of paper and, you know, here, write down what your needs are.  It has to be relevant, and meaningful. | Because they don't expect to get those needs fulfilled.  I think, some work on encouraging them, that, we’re in an environment now where things can change, and we would like to know.  But I think one of the problems is making people feel like they can state their preferences  So I think to create an environment where people do feel confident in stating their preferences  It's the information needs to be meaningful; it needs to be relevant and meaningful to the patient. I think that information needs to be prepared in such a way, and education is to be prepared in such a way, that the patients actually understand.  I am sure most patients are more than happy and wouldn’t be offended and wouldn’t think why don’t you already know this | The cultural norms that pervade existing healthcare services, including palliative care and primary care, preclude those from different cultures accessing these services.  People from diverse cultural backgrounds (not White British) may have low expectations around how the services may or may not meet their needs (modifiable context). Systemic power imbalances exist that make it difficult / impossible for people to explain how / why a service does not fit their needs (m). As a result people will “look after themselves” (o) and / or their needs will not be fulfilled.  Systemic power dynamics (c) require that patients comply with certain norms and act as “a good patient” (m). In this position, patients are disempowered (m) and negotiating appropriate, culturally sensitive care according to culture is impossible (o).  Culture and beliefs influence every aspect of healthcare – the extent of this (e.g. rituals around taking medication) require much more attention. (Unknown unknowns)  Patients and carers are required to “go in” to healthcare environments (c) – adding to disempowerment (m).  There are language barriers (c). |
| **CULTURALLY COMPETENT CARE STARTS WITH…**  **CULTURALLY COMPETENT LEADERSHIP**  white privileged people need to be allies to our non-white, non-cis, non-het, non-whatever else, you know, colleagues  **CULTURALLY COMPETENT TRAINING**  the really urgent need for more cultural competency training across health and social care, and how underrepresented both ethnic minorities and then also people from lower socioeconomic backgrounds…  the vast majority of the specialist palliative care workforce is White British, particularly our specialist nursing service. .    And it needs, almost, to be completely flipped on its head, doesn't it, with the community development workers doing the education in the specialist services, and then out again.  I don't know what you think about that? | To support them in becoming health and social care professionals, so that goes to things like mentoring potential medical students from different cultural groups.  So, there's a risk that we would be just propagating some of those inequalities if the White British workforce were delivering that education into primary care, for example | to give them a shove up the ladder  he did spiritual care plans, which you’re supposed to think about as frequently as you do an actual care plan which was brilliant, and then he did online learning modules about what needs to be in the environment for people of different cultural backgrounds when it comes to end of life care and in fact any sort of fragile care of a child. | Multi-cultural leadership is an important context for enabling more culturally competent palliative care. This will require increased self-awareness amongst White British leaders (the majority)(m) and professional attitudes (m) to allow the development of leaders from a wide range of backgrounds at all levels of the NHS, including in frontline practice (allies)  There is an urgent need for more training in culturally competent healthcare / palliative care.  Spiritual / cultural care planning |
| **CULTURALLY COMPETENT CARE – WORKFORCE**  the lack of diversity in the specialist palliative care workforce,  a very white, very middle-class profession and there’s an obvious knock-on from that.  if we’re thinking of improving or changing the system so that a community in [place], for example, might have district nurses of different backgrounds and perhaps, I don’t know, take it from there? | we want people from our own culture to meet our needs and take care of us.  she can feel that sense of relief. It’s something she can feel, just an instant connection and understanding of spoken and unspoken cultural and language needs  really, really helped that she just walked into our house and immediately understood that we were probably doing things slightly differently  to get better in our whiteness of not being worried to ask people what they need and to put our hands up, you know, I’m not an expert in your culture | oh, thank goodness, here’s somebody who’s going to get it. We can probably apply it to all cultures, couldn’t we? And that general feeling of do we get palliative care or not?  and we felt very relaxed with her  Please could you tell me what would make your care really great? And what do I need to know about you and your family and the care that you need? | Having someone from that  person’s own background, there’s  often a sense of relief about that in  terms of being understood but it was  also pointed out that we can’t really  leave that to the people of those  backgrounds, that we all actually  have to be really aware and alert to  those issues and working hard to try  and resolve those problems.  Underlying mechanism of self awareness  Needs to be supported by context / culture |
| **INTEGRATION** | | | |
| **INTEGRATION / COMMUNICATION TEAMWORKING**  **Non-negotiable:**  **Increased demand due to COVID-19**  breaking down of barriers  **Negotiable**  **Environment that facilitates cross-boundary communication**  If the communication in its widest sense could be accessible,  **Decreased bureaucratic processes**  it’s meant that we’ve avoided things like formal referrals into services, patients haven’t been on waiting lists for things  **Co-location**  disjointedness of the services  bringing in many other specialists under the same roof  district nurses or the community nurses have been removed from their surgery  relationships between GPs and district nurses depend on relationships from a very, very long time ago | they feel like they’re part of a team  Mechanisms = shared vision, collective buy-in, removing threat, ability to change and innovate (reject the way it’s always been)  builds our trust and enables us to do it again as a team the next time  and they feel like they’re making a real difference.  Positive reinforcement – trust, faith in systems and colleagues  where they can easily meet, talk (team) | **Patients**  helping those families to have as normal an experience as possible and really having that holistic approach,  easy to gain what you need at that time  can coordinate and communicate and collaborate well and do things, it will at least solve one of their so many problems they are going through  as a patient you should almost not notice it’s going on and that would signify that it’s a successful collaboration, but you’ve done a really good job of pointing out, yes, what the other benefits are kind of further down the line and for the professionals involved I think as well, which ultimately benefit the patients in the end as well.  Staff  Professionals can just talk to each other, agree who the most appropriate person is to meet whatever the presenting need is at that time and then sort it out there and then.  more satisfying, rewarding way of working,  and then solve problems in palliative care.  established when the ways of working were different and the district nurses often sat in offices in the GP surgery, so a lot of the informal conversation could happen. | The rapid increase in need for community end-of-life care that occurred during the COVID-19 pandemic (c) triggered shared vision (m) amongst primary care professionals leading to collective buy-in (o) and commitment to the provision of end-of-life care in the community for patients and families (o).  During COVID-19, there was an ability for professionals in clinical practice and service management to reject the status quo (c), as well as a decrease in bureaucratic processes (c), both of which allowed innovation (o) and a sense that longstanding barriers to collaborative working were suspended (m).  Positive experiences of providing end-of-life care collaboratively (o) through effective communication and co-ordination (c) lead to an increase in trust between professionals (m). This positive experience enables confidence in collaborative working to deliver palliative and end-of-life care (o) and provided a more satisfying and rewarding way of working (o).  Collaborative, effective teamwork in end-of-life care (o) is further enabled by close co-location of professionals (c), enhancing the communication and positive experience required to ensure trust and confidence in the team relationship (m). |
| **RELATIONSHIPS**  **Patient-professional**  **(Unequal power dynamics)**  clinicians do find it difficult, especially when they’re trying to do what they think is best for us.  partnership behaviour and the group that I was in talked about it’s about relationships not systems  part of that process of listing them [their needs] with the patient, try to prioritise which ones not only are important to them, but which ones can be solved most quickly, or most easily.  Because I can imagine people could have needs that are maybe very difficult to solve.  They may be very important to them, but if they can't be easily tackled, it might be worth going down the list to the next most important  the relationship between a patient and their clinician can be compromised when we try to do that shoehorning into one category or another and if things don’t work out the way it’s sort of magically been predicted by a clinician then trust can be compromised  **Professional**  individual relationships and how they can be very different depending on people’s motivations.  **Specialist-GP**  there’s often tension in that relationship  my consultations about the relationship with GP community and if the GP, they say they’re great, I kind of relax a little, I suppose, and sort of think, okay, this is going to go well, we’ve got a team approach here. But if they say, oh, no, I’ve never seen my GP or have real issues | of those awkward patients who says no and asks questions.  there's a real skill, I think, and I think we have it across palliative care, and primary care, I'd like to think, is in holding that kind of uncertainty, and almost insolvability with a person  individual effort, you know, real determination, pushing through a system that perhaps doesn’t make it that straightforward to do these things and…  Leadership  then who would take ownership  level of expertise, personal commitment, trust, ability to bear witness  depending on people’s motivations  do I just do what is needed to try and make sure it works?  knowledge enough to understand palliative care needs, | And if you knock one thing off the list, it might make the other things seem a bit better, anyway.  making the best possible decision around their care,  tendency of thinking, well, can I change that? Can I change that dynamic in this situation or for this person in front of me  the relationship complicated sometimes because then that becomes the expectation of the primary care team when in fact it’s their job. | There are specific skills required for palliative and end-of-life care consultations including an ability to hold uncertainty (m) and accept that some patient problems cannot be “solved” (m). Effective palliative and end-of-life care depends on relationships between patients and professionals (c) where there (often unspoken) is mutual understanding of the situation (m) and trust (m) so that patients and carers are able to describe their distress / ask the awkward questions (o). This can be difficult to deliver in a fragmented, single-disease, solution-focussed healthcare system (c). When patients and carers feel their distress is unaddressed (m), or that they have been “shoehorned” into a solution (m), they lose confidence and trust, and their distress can rise (o). Equally when a professional witnesses a failure in the solution they have proposed (from a medical paradigm) this can cause frustration and distress (m) and compromise the relationship with the patient. The approach varies between professionals and at different times depending on their personal motivation, values and conflicting demands on their time.  Interprofessional relationships between specialist palliative care colleagues and general practitioners are variable (o). There can be tension in the relationship.  Professionals from Specialist Palliative Care feel other services do not deliver the care that a person needs (? Related to expertise, approach and resource) / experience inadequacy in other services (c) leading to a feeling of responsibility / obligation (m) to deliver this care. Primary care professionals who experience this level of service from palliative care will come to expect it (m). This can lead to a fragile relationship with primary care (o) with uncertain roles and a lack of trust. A negative cycle follows with one suboptimal experience framing expectations for the next (o). *This is all further complicated by funding*  *Responsibility taking – “I just need the GP to come”* |
| **PERSONAL MOTIVATION OF STAFF**  a district nurse and she really enjoyed her job, her speciality being terminal care.  what words I use on my referral form to put a case for need about why I think the district nurses need to be involved. It’s much more trickier if there’s not a specific task that needs doing | very fulfilling  individual motivation of service providers and GPs, practitioners, around the motivation for integration because if you’re pretty switched on to palliative care, if it’s something that you really care about  district nurse referrals myself and it can vary how accepting they are  intuition that things may not go well.  individual relationships and how they can be very different depending on people’s motivations. |  | Contexts = under-resourced, task orientated fragmented system. Trust not a given. Commitment to palliative care not a given |
| **MDT**  employed care coordinators  coordinator being skilled  social prescribers or link workers,  GP should be that care coordinator  the primary care networks  many allied healthcare professionals now coming in to help you GPs, the physician assistants, green prescribers, the link workers  more honest conversations around professional attitudes towards palliative care    training was there and the space for  professionals to get together and  talk about how palliative care works  for their patients individually | patient advocates and care coordinators and skilled communicators,  so it becomes embedded in everything we do  so more practitioners would be more inclined to do it better if it's easy for them to do.  +++ Positive feedback | **Patients and carers**  no wrong door and that people should be able to approach one person and whoever they make that approach to should be able to link them in to the care that they need.  of coordinating. And coordination, what does that mean? It means communication and collaboration, and giving as much information to the significant other of how that palliative care is going, so that we are not only relieving them from another cycle of emotional distress, because the family member is already in that stage, it’s too much for them to handle  more likely to forge those relationships in order to make things work,  and actually that it empowers people to do it better.  [relationships are really important because it] makes it much easier for the whole palliative care process to work, not only with carers and families but also with other professionals  **Staff / patients**  and gain those benefits from partnership working  . | A key factor in effective co-ordination of care is the multi-disciplinary team (c). For each individual patient this is made up of a range of professionals from different organisations (c).  Within the team there is a need for an identified individual who can co-ordinate the MDT and act as an advocate for the patient. This role often falls to carers, who are vulnerable to emotional distress (o).  A range of professionals have the potential to be an identified care co-ordinator role within the MDT (GP, allied healthcare professional, social prescriber) (c). In order to do so they require personal values and commitment to the palliative care approach to care (m). Trusted (m) relationships with other team members and the patient / carers (c) are also critical in order to deliver an appropriate and timely response to care needs (o).  Positive experiences of care co-ordination and MDT working (e.g. fulfilment, job satisfaction *need more work to understand further)* for professionals, carers and patients provide positive feedback and affirmation (m), growing confidence (m) and driving a commitment (m) to similar work in the future (o*). (+ ? role modelling*)  Team relationships can be built during events such as training and education (c) where professionals share common challenges (m) and develop shared solutions. |
